# Cannabis use and symptom severity in individuals at ultra high risk for psychosis: a meta‐analysis

**DOI:** 10.1111/acps.12699

**Published:** 2017-02-07

**Authors:** R. Carney, J. Cotter, J. Firth, T. Bradshaw, A. R. Yung

**Affiliations:** ^1^ Division of Psychology and Mental HealthUniversity of ManchesterManchesterUK; ^2^ Division of NursingMidwifery and Social WorkUniversity of ManchesterManchesterUK; ^3^ Greater Manchester West Mental Health NHS Foundation TrustManchesterUK

**Keywords:** cannabis, ultra high risk, clinical high risk, at‐risk mental state, substance use, prodrome

## Abstract

**Objective:**

We aimed to assess whether individuals at ultra high risk (UHR) for psychosis have higher rates of cannabis use and cannabis use disorders (CUDs) than non‐UHR individuals and determine whether UHR cannabis users have more severe psychotic experiences than non‐users.

**Method:**

We conducted a meta‐analysis of studies reporting cannabis use in the UHR group and/or positive or negative symptoms among UHR cannabis users and non‐users. Logit event rates were calculated for cannabis use, in addition to odds ratios to assess the difference between UHR and controls. Severity of clinical symptoms in UHR cannabis users and non‐users was compared using Hedges’ g.

**Results:**

Thirty unique studies were included (UHR *n* = 4205, controls *n* = 667) containing data from cross‐sectional and longitudinal studies, and randomised control trials. UHR individuals have high rates of current (26.7%) and lifetime (52.8%) cannabis use, and CUDs (12.8%). Lifetime use and CUDs were significantly higher than controls (lifetime OR: 2.09; CUD OR: 5.49). UHR cannabis users had higher rates of unusual thought content and suspiciousness than non‐users.

**Conclusion:**

Ultra high risk individuals have high rates of cannabis use and CUDs, and cannabis users had more severe positive symptoms. Targeting substance use during the UHR phase may have significant benefits to an individual's long‐term outcome.

**Summations**

- Ultra high risk (UHR) individuals are more likely to have used cannabis in their lifetime than controls.
- UHR individuals are more likely to have a comorbid cannabis abuse disorder than controls.
- UHR cannabis users had significantly higher levels of unusual thought content and suspiciousness compared with UHR non‐cannabis users.

**Considerations**

- Included studies often had different methods of identifying cannabis users, and in some cases, use of cannabis was not a primary outcome but was reported as a mediating variable, or secondary area of interest.
- We were unable to account for the use of other substances in our meta‐analysis which may have driven the relationship between cannabis use and increased severity of specific positive symptoms.
- Substantial heterogeneity was observed, which may have been the result of clinical and/or methodological differences across studies.

## Introduction

Cannabis is one of the most frequently used illicit drugs worldwide 1. It significantly increases the risk of developing a psychotic disorder, particularly among those individuals who use it at an early age 2, 3, 4, who frequently use high‐potency cannabis or ‘skunk’ 5, 6 and who have a genetic predisposition for psychosis 7.

People with schizophrenia are more likely to use cannabis and have comorbid substance use disorders than the general population 8. This increased comorbidity is associated with poor clinical outcomes: increased premature mortality, poor engagement with services and increased rates of hospitalisation 9, 10. Given the risks of continued substance use, it is important to identify when these problems first emerge. High rates of cannabis use are often observed at an early stage, in people with first‐episode psychosis (FEP; 11). Yet this unhealthy profile may even predate the onset of full psychotic symptoms, during the ultra high risk (UHR) phase.

Young people at UHR for psychosis (also referred to as ‘clinical high risk (CHR)’ or ‘at‐risk mental state’) can be identified using operationalised criteria 12, 13, 14. An individual must fit one, or a combination of the following criteria: presence of attenuated psychotic symptoms, brief intermittent psychotic symptoms or a genetic‐risk combined with a recent decline in functioning 15. Substance use research in the UHR group has mainly focussed on identifying whether cannabis use predicts transition to psychosis. A recent meta‐analysis provides evidence for a dose–response relationship, where heavy cannabis use (including abuse or dependence) predicted increased likelihood of later transition to psychotic disorder 16.

Previous reviews have also explored the prevalence of substance use in the UHR group, 17, 18. However, the findings of these reviews were largely inconclusive, due to the lack of research available when the searches were conducted, and the main conclusions were that more prospective studies are required before any conclusions can be made regarding substance use in this group. To date, no meta‐analyses have been conducted in this area to allow for more robust conclusions, and a meta‐analysis comparing cannabis use in the UHR group to healthy controls (HCs) is lacking. Additionally, little is known about the relationship between cannabis use and attenuated positive and negative symptoms in the UHR group. This is despite many studies reporting a link between symptom severity and cannabis use in FEP and schizophrenia 19. For example, FEP individuals who used cannabis had more severe positive symptoms including hallucinations, suspiciousness and delusions, in addition to other clinical factors such as mania and poor general functioning than non‐cannabis users 19.

Thus we aimed to provide robust, up‐to‐date statistical analyses of the literature examining cannabis use in the UHR group. Therefore, this review aimed to address the following questions:

- Do UHR individuals have higher rates of current and lifetime cannabis use than HCs?
- Do UHR individuals have higher rates of cannabis use disorders (CUDs) than HCs ?
- Do UHR cannabis users have higher positive and negative symptoms than non‐cannabis using UHR subjects?

## Method

This review was conducted according to PRISMA guidelines for reporting systematic reviews 20.

### Study inclusion

Eligible studies were original research articles published in peer‐reviewed journals, with populations meeting criteria for being at ‘UHR’ or ‘CHR’ (or similarly defined) of psychosis, based on a clinically recognised instrument 21. Studies reporting the proportion of UHR individuals who claimed to currently use cannabis, or have done so in their lifetime, or having a current/lifetime CUD in accordance with DSM‐IV/ICD criteria were included. Studies were also included if they reported positive or negative symptoms in both UHR cannabis users and non‐users, as measured by a clinically validated tool. Eligible studies included cross‐sectional and longitudinal analyses or intervention studies reporting baseline data on cannabis use in UHR individuals.

Studies including only subjects at genetic‐risk, case studies, reviews and non‐English language articles were excluded. Studies reporting general substance use and not cannabis use specifically were also excluded. Where participant samples overlapped, only the larger sample was included in the review. Where study samples overlapped but reported different outcomes, for example cannabis use and cannabis dependence, both were included in the respective meta‐analyses. Authors were contacted if it was unclear whether samples overlapped. To avoid bias, studies using UHR individuals recruited solely from prisons, or young offender institutions were excluded 22 as substance use in these groups tends to be higher than in the general population 23.

### Search strategy

On 8th December 2016, we conducted an electronic database search of Ovid MEDLINE, PsycINFO, EMBASE, AMED and the Cochrane Central Register of Controlled Trials (CENTRAL) using the following keyword search terms: [‘clinical high risk’ or ‘CHR’ or ‘ultra high risk’ or ‘UHR’ or ‘at‐risk mental state’ or ‘ARMS’ or ‘attenuated positive symptoms’ or ‘attenuated psychotic symptoms’] and [‘psychosis’ or ‘psychotic’ or ‘schizophrenia’] and [‘cannabis’ or ‘marijuana’ or ‘substance use’ or ‘substance abuse’ or ‘substance dependence’ or ‘substance misuse’ or ‘drug*’ or ‘recreational drug’ or ‘drug abuse’ or ‘hallucinogen’]. In addition, a basic search of Google Scholar was conducted and the reference lists of retrieved papers were reviewed to identify any additional relevant publications.

### Study selection and data extraction

Three authors (R.C, J.F & J.C) independently screened articles for eligibility. A tool was developed to extract the following data for eligible studies: 1 study characteristics (author, year of publication, country of origin, study design); 2 sample demographics (sample size, gender composition, mean age); 3 instrument used to assess at‐risk status; 4 rate of cannabis use in UHR and control groups (measure, prevalence or sample mean); 5 ICD/DSM CUDs (measure, prevalence); 6 positive and negative symptoms for UHR cannabis users and non‐cannabis users (sample size, measure, mean, standard deviation); 7 summary of findings. Studies that included a HC group were assessed for quality using the Newcastle–Ottawa Scale 24, a validated instrument for non‐randomised trials and observational studies. The scale utilises a star system to assess selection of participants, comparability of groups and assessment of outcome or exposure of interest. Studies awarded 8–9 stars were classed as high quality, 4–7 medium quality and 0–3 low quality. Any disagreements were resolved through discussion.

### Statistical analysis

All statistical analyses were performed using comprehensive meta‐analysis Version 3.0 25. Proportional meta‐analyses using random‐effects models were used to estimate logit event rates of current and lifetime cannabis use across the UHR samples. To assess the difference in cannabis use between UHR and HCs, odds ratios were used, and 95% CI were calculated. Standardised mean differences (SMD) were calculated to assess differences in overall positive and negative symptom severity between UHR cannabis users and non‐users using Hedges’ g. SMDs were also conducted on individual positive symptoms if reported among three or more samples. Random‐effects models were used throughout to account for heterogeneity between studies 26, 27. Heterogeneity across studies was quantified using the *I* ^2^ statistic 28.

## Results

### Study characteristics

**Figure 1:**
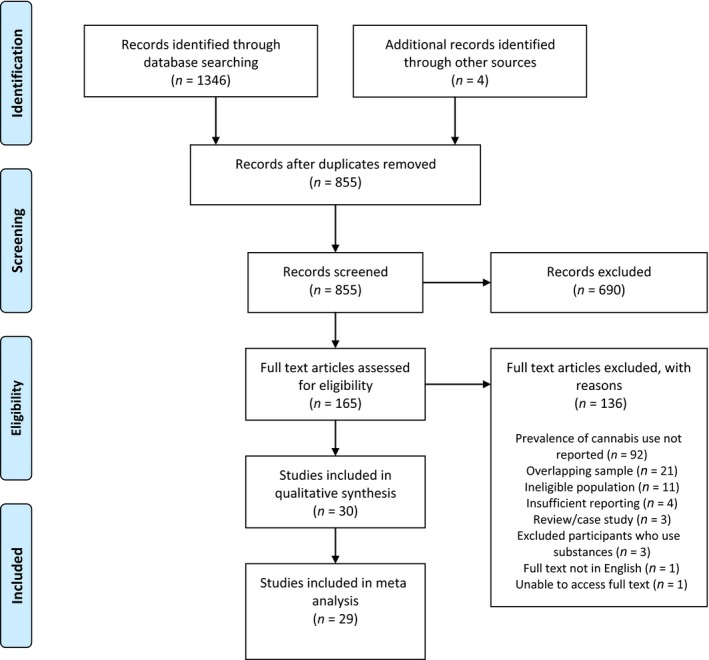
PRISMA flow diagram. [Colour figure can be viewed at wileyonlinelibrary.com]

The study selection process is summarised in Fig. 1. A total of 30 unique citations were included (UHR *n* = 4205; controls *n* = 667): 26 studies from the initial search and four additional studies from searching of reference lists (Table 1). Studies were conducted in 10 countries: Canada (*n* = 6), Netherlands (*n* = 5), USA (*n* = 5), Switzerland (*n* = 3), Austria (*n* = 2), Australia (*n* = 4), UK (*n* = 2), France (*n* = 1), Germany (*n* = 1) and Spain (*n* = 1). Study samples overlapped in three instances; however, different outcomes were included in separate meta‐analyses 29, 30, 31, 32, 33, 34. The majority of studies that included a control group were deemed medium quality, with only one study rated as high quality 35, (see Appendix S1 for individual scores).

**Table 1:** Studies included in review

Study reference and country	Group	*N* (male/female)	At‐risk screening instrument	Outcome of interest	Study design
Addington et al. 35 – Canada	UHR	360 (211/149)	SIPS	Current cannabis use, DSM‐IV current/lifetime cannabis abuse	Longitudinal
	HC	108 (87/21)			
Amminger et al. 29 – Austria	UHR	81 (27/54)	PANSS/GAF	Current cannabis use	RCT
Amminger et al. 30 – Austria	UHR	69^a^ (27/54)	PANSS/GAF	DSM‐IV cannabis abuse disorder	RCT
Auther et al. 32 – USA	UHR	101 (66/35)	SIPS	Current/lifetime cannabis use, DSM‐IV cannabis abuse, SIPS‐positive/negative symptoms in cannabis users	Longitudinal + cross‐sectional
	HC	59 (30/29)			
Auther et al. 31 – USA	UHR	341 (210/131)	SIPS	Current cannabis use, DSM‐IV cannabis abuse disorder, SIPS‐positive/negative symptoms in cannabis users	Longitudinal + cross‐sectional
Bechdolf et al. 57 – Germany	UHR	156 (106/50)	SIPS	DSM‐IV cannabis abuse disorder	RCT
Bloemen et al. 58 ‐ Netherlands	UHR	37 (26/11)	SIPS	Current/lifetime use of cannabis	Longitudinal
	HC	10 (8/2)			
Bousman et al. 33 – Australia	UHR	225 (93/132)	CAARMS	Lifetime cannabis use	Longitudinal
Buchy et al. 59 – Canada	UHR	735 (423/312)	SIPS	Current/lifetime cannabis use, DSM‐IV cannabis abuse or dependence, SIPS‐positive/negative symptoms in cannabis users	Longitudinal
	HC	278 (140/138)			
Buchy et al. 60 – Canada	UHR	170 (96/74)	SIPS	Current cannabis use, DSM‐IV cannabis abuse or dependence	Longitudinal
Bugra et al. 61 – Switzerland	UHR	74 (47/27)	BSIP	Current/lifetime cannabis use, BPRS positive and SANS negative symptoms in cannabis users	Cross‐sectional
Carney et al. 36 – Australia	UHR	279 (93/186)	CAARMS	Current/lifetime cannabis use	Cross‐sectional
Corcoran et al. 62 – USA	UHR	32 (26/6)	SIPS	Lifetime cannabis use, DSM‐IV cannabis abuse or dependence, SIPS‐positive/negative symptoms in cannabis users	Longitudinal
Dragt et al. 63 – Netherlands	UHR	243 (140/103)	SIPS/BSABS‐P	Lifetime cannabis use, DSM‐IV cannabis use disorder, SIPS‐positive/negative symptoms in cannabis users	Longitudinal
Gill et al. 37 – USA	UHR	102 (79/23)	SIPS	Current cannabis use, SIPS‐positive symptoms in cannabis users	Cross‐sectional
Hagenmuller et al. 64 – Switzerland	UHR	86 (53/33)	SIPS	Current cannabis use	Cross‐sectional
	HC	47 (23/21)			
Machielson et al. 65 – Netherlands	UHR	59 (52/7)	SIPS	DSM‐IV cannabis abuse or dependence, SIPS‐positive/negative symptoms	Cross‐sectional
Magaud et al. 66 – France	UHR	77 (92/46)	CAARMS	Current cannabis use	Cross‐sectional
Marshall et al. 67 – Canada	UHR	48 (33/15)	SIPS	DSM‐IV cannabis abuse disorder	Longitudinal + cross‐sectional
McHugh et al. 38 – Australia	UHR	190 (76/114)	CAARMS	Lifetime cannabis use	RCT
Mizrahi et al. 68 – Canada	UHR	24 (13/11)	SIPS	SIPS‐positive/negative symptoms in cannabis users	Cross‐sectional
Nieman et al. 69 – Netherlands	UHR	147 (71/76)	CAARMS	Current cannabis use, CAARMS‐positive/negative symptoms	Cross‐sectional
Phillips et al. 34 – Australia	UHR	100 (49/51)	CAARMS	Lifetime cannabis use, DSM‐IV cannabis dependence	Longitudinal
Pruessner et al. 39 – Canada	UHR	30 (16/14)	CAARMS	Current cannabis use	Cross‐sectional
	HC	30 (15/15)			
Russo et al. 70 – UK	UHR	60 (31/29)	CAARMS	Current/lifetime cannabis use	Cross‐sectional
	HC	60 (26/34)			
Simon & Umbricht 71 – Switzerland	UHR	72 (43/29)	SIPS	Current cannabis use	Longitudinal
Stojanovic et al. 72 – Spain	UHR	17 (12/5)	CAARMS	Current cannabis use	Cross‐sectional
	HC	25 (12/13)			
Valmaggia et al. 40 – UK	UHR	182 (104/78)	CAARMS	Current/lifetime cannabis use	Longitudinal
Van Tricht et al. 73 – Netherlands	UHR	48 (32/36)	SIPS	SIPS‐positive/negative symptoms in cannabis users	Cross‐sectional
	HC	50 (33/17)			
Woods et al. 41 – USA	UHR	60 (39/21)	SIPS	Lifetime cannabis abuse or dependence	RCT

HC, healthy controls; BPRS, brief psychiatric rating scale; BSABS, bonn scale for the assessment of basic symptoms; BSIP, basel screening instrument for psychosis, CAARMS, comprehensive assessment of at‐risk mental states; DSM‐IV, Diagnostic and statistical manual of mental disorders IV; SIPS, structured interview for prodromal symptoms; UHR, ultra high risk; PANSS, positive and negative syndrome scale; RCT, randomised controlled trial.

a Long ‐term follow‐up, missing data for 12 participants.

### Current and lifetime cannabis use

**Figure 2:**
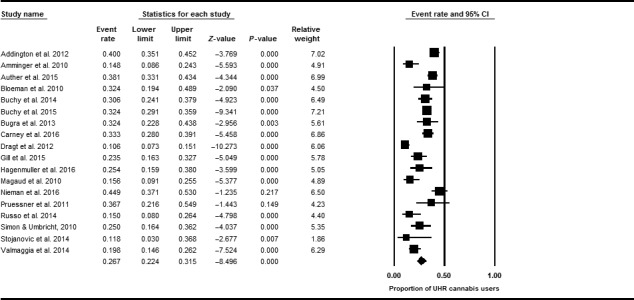
Summary of rates of current cannabis use in ultra high risk individuals.

Eighteen studies stated the proportion of UHR individuals who self‐reported current cannabis use, defined as any use within the past month, with the exception of one study 39, which defined current use as any cannabis within the past 3 months. Proportionate meta‐analysis revealed that 26.7% of UHR individuals currently used cannabis (*n* = 3068, 95% CI = 0.22–0.32; *I* ^*2*^: 85.70%; Fig. 2). A sensitivity analysis removing the study which defined current use as cannabis intake within the last 3 months (rather than last month) found that excluding this study had a negligible impact on the results. Comparisons of current cannabis use in UHR and non‐UHR control groups indicated that UHR individuals were more likely to be current cannabis users than HCs, although the difference fell short of statistical significance (OR: 1.56; *P* = 0.08; 95% CI: 0.94–2.57; *I* ^2^: 59.52%; see Appendix S1).

**Figure 3:**
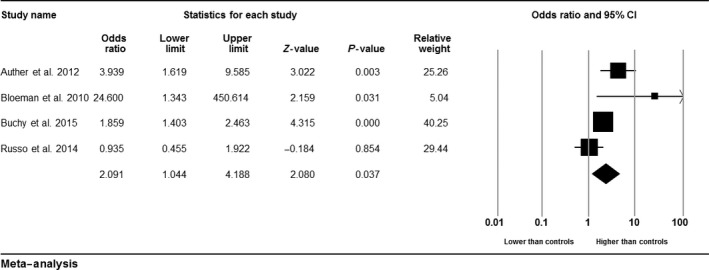
Lifetime use of cannabis ultra high risk vs. healthy controls.

Eleven studies reported lifetime cannabis use. Proportional meta‐analyses found that 52.8% of UHR individuals had used cannabis at some point in their lifetime (*n* = 2251, 95% CI = 0.47–0.59; *I* ^*2*^: 84.02%). UHR individuals were also significantly more likely to have used cannabis in their lifetime compared with HCs (OR: 2.09; *P* = 0.037; 95% CI: 1.04–4.19; *I* ^2^: 67.63%; Fig. 3).

### Cannabis use disorders (CUD)

Eleven studies reported comorbid cannabis abuse or dependence disorders (CUD) in UHR individuals, according to the DSM‐IV criteria. Meta‐analysis of prevalence rates indicated 12.8% of UHR individuals had a current comorbid CUD (95% CI = 0.09–0.19; *I* ^*2*^: 90.32%). UHR individuals were significantly more likely to have a CUD than controls (OR: 5.49, *P* = 0.001; 95% CI: 1.97–15.32; *I* ^*2*^: 0%). Lifetime CUDs were reported in only two studies and were not included in the meta‐analysis 35, 41. The rates of lifetime CUDs were 12.8% (*n* = 46) and 26.7% (*n* = 16) respectively.

### Positive and negative symptoms

Table 2 displays the effect and sample sizes, heterogeneity statistics and significance values of the relationships between cannabis use and symptoms in UHR individuals.

**Table 2:** Meta‐analyses outputs

Cannabis use in UHR individuals	Studies Included (*k)*	N (UHR)	Event Rate	95% CI	I^2^	‐	‐
Current cannabis use	18	3068	0.267	0.22–0.32	85.70		
Lifetime cannabis use	11	2251	0.528	0.47–0.59	84.02		
Current cannabis abuse disorder	11	2315	0.128	0.09–0.19	90.32		

UHR vs. HC	Studies Included (*k)*	*N* (UHR)	*N* (HC)	Odds Ratio	95% CI	*I* ^2^	*Z*	*P*‐value
Current cannabis use	7	1289	622	1.56	0.94–2.57	59.52	1.73	0.08
Lifetime cannabis use	4	930	405	2.09	1.04–4.19	67.63	2.08	0.04
Current cannabis abuse disorder	2	1095	458	5.49	1.97–15.32	0	3.25	0.001

Symptoms in UHR‐CU vs. UHR‐NU	Studies Included (*k)*	*N* (UHR‐C)	*N* (UHR‐NC)	Hedges’ g	95% CI	*I* ^2^	*Z*	*P*‐value
Total Positive Symptoms	8	325	593	0.16	‐0.05–0.37	45.70	1.52	0.13
Disorganised Speech	4	244	452	0.05	‐0.27–0.38	71.05	0.31	0.75
Grandiosity	3	178	371	0.11	‐0.11–0.32	19.96	0.96	0.34
Perceptual Abnormalities	4	244	452	0.05	‐0.115–0.206	0.00	0.56	0.57
Suspiciousness	3	178	371	0.21	0.02–0.39	0.00	2.22	0.03
Unusual Thought Content	4	244	452	0.27	0.07–0.48	30.29	2.63	0.01
Total Negative Symptoms	7	301	515	‐0.03	‐0.29–0.23	59.77	‐0.23	0.82

UHR, ultra high risk.

Scores for total positive symptoms were derived predominantly from overall scores on the positive items of the SIPS 42. Total positive symptoms did not significantly differ between UHR cannabis users and non‐cannabis users. In two studies, UHR cannabis users also included those who had used in their lifetime and removal of these studies did not affect significance levels. When individual items of positive symptom scales were analysed, UHR cannabis users were found to have significantly higher levels of unusual thought content (UTC) and suspiciousness than non‐cannabis users, but no differences were found for perceptual abnormalities, grandiosity or levels of disorganised speech (Table 2). Negative symptoms were reported less frequently, and no significant difference was found for overall scores between cannabis users and non‐users.

## Discussion

This meta‐analysis indicates that UHR individuals have high rates of cannabis use. They are more than twice as likely to have used cannabis in their lifetime compared with HCs. UHR individuals were also more than five times as likely to have a current cannabis abuse disorder compared to HCs. This is particularly problematic given the risks associated with continued cannabis use. Even prior to the onset of psychotic disorders, cannabis use is associated with increased severity of certain positive symptoms, as UHR cannabis users had significantly higher levels of unusual thought content and suspiciousness compared with non‐users.

### Cannabis use in UHR

We found that 52.8% of UHR individuals reported using cannabis in their lifetime, which is a similar proportion to FEP samples 11, and higher than that of healthy populations 43. Indeed, our analyses found significantly higher rates of lifetime cannabis use in the UHR samples than in the HC groups. Meta‐analyses also revealed approximately one in four UHR individuals currently used cannabis. We also found high rates of comorbid CUDs in UHR individuals (12.8%). This is slightly lower than that found in people with schizophrenia (16%) 8. However, it is important to consider that we focused on young people in the UHR phase; that is, those who are putatively prodromal and are not yet experiencing full psychotic symptoms. Therefore, even prior to the onset of psychosis, UHR individuals are likely to engage in risky cannabis use.

High rates of cannabis use in this group are perhaps unsurprising given that use of substances is common in young people who present for mental health care 44, 45 and people with early psychosis 11. As there is evidence to suggest frequent use of high‐potency cannabis increases the risk for later transition 5, 6, it is important that early intervention services encourage substance use reduction upon first presentation. A previous review and meta‐analysis found that UHR individuals are significantly more likely to smoke, abuse alcohol and have lower levels of physical activity than their peers 46. Here, we add to this evidence to suggest that this group is also more likely to have used cannabis or have a CUD, posing an additional risk factor to both physical and mental health.

### Cannabis use and symptoms

Our meta‐analysis is the first to find a statistically significant association between UHR cannabis use and more severe positive symptoms (unusual thought content and suspiciousness). This is in line with previous research in people with FEP. For example, the use of cannabis at the time of, and after FEP, is associated with increased positive symptoms and poorer psychosocial functioning and long‐term outcome 10, 19. It also supports the findings of Valmaggia et al. 40 where UHR participants often reported that they stopped using cannabis due to exacerbation of positive symptoms. Similar to Seddon et al. (2016), we also found no association between cannabis use and negative symptoms. We were unable to analyse individual negative symptoms due to a lack of available data. As such, analysis of global symptom domains may have masked any differences in individual symptoms.

We can only speculate about the reasons for the association between cannabis and increased positive symptoms. Positive symptoms may occur as a direct result of substance use. Indeed, cannabis can induce symptoms of psychosis in healthy populations, and may therefore influence symptom severity in the UHR group 47. Alternatively, those with more pronounced positive symptoms may be more likely to self‐medicate using substances such as cannabis 48. However, a study by Gill et al., 37 found that mood enhancement was the primary reason for cannabis use reported by UHR individuals. Therefore, it could be used as a way to alleviate other symptoms such as anxiety or low mood 45 that are frequently found in the UHR group 49, 50. Another possibility is that a separate factor is driving the increase in positive symptoms. Our meta‐analysis does not take into account potential confounders, such as alcohol or other substance use. Cannabis users are significantly more likely to engage in use of other substances, which may contribute to severity of positive symptoms 51. For example, a recent cohort study found alcohol confounds the relationship between cannabis use and transition to full‐threshold psychotic disorders 31. We were also unable to account for the last time a person used cannabis across all studies. Therefore, increased positive symptoms could be due a result of the acute, intoxicating effects of cannabis.

From the studies included in our analysis, we were unable to account for the strength of cannabis young people were using. This may have been why we only found a significant difference for two of the positive symptoms. People with psychosis are more likely to use high‐potency cannabis or ‘skunk’ 52. As high‐potency cannabis has been shown to have the most harmful effects for both mental and physical health, it may be that those using the strongest forms of cannabis experience more severe symptoms. Similarly, the adverse health effects of synthetic cannabinoids such as ‘spice’ have been recognised, with the increased risk for psychotic‐like experiences being a primary area of concern 53. As there has been a recent rise in the use of synthetic cannabinoids, more research is required to establish the effect these have on mental health as well as the patterns of use in people with emerging mental health difficulties.

### Clinical implications

Irrespective of causation, high rates of cannabis use in the UHR group carries important clinical implications. Although many UHR individuals will not develop full‐threshold psychosis, they may go on to have anxiety, mood or substance use disorders 50, and continue to function poorly regardless of transition or symptomatic remission 54, 55. Therefore, it is important to address any comorbid disorders at an early stage. Future research should assess the efficacy of interventions used to reduce cannabis use in UHR individuals upon first presentation to mental health services. For example, motivational interviewing and cognitive behaviour therapy have been found to be effective in reducing cannabis use among early psychosis groups 57, although a randomised control trial in the UHR group is yet to be conducted. Longitudinal studies are also required to highlight any relationship between continued cannabis use and factors such as long‐term outcome, functioning and symptoms over time.

### Limitations

High levels of heterogeneity were observed for all estimates which likely reflect clinical and methodological differences between studies. We performed sensitivity analyses in which we removed each study in turn and found that this had a negligible impact on the heterogeneity statistics, indicating that the *I* ^2^ values were not the product of the inclusion of a single study but instead reflected wider between‐study differences. These may have been driven by different recruitment strategies, study locations, sample demographics and instruments that were used to define and report substance use. We included studies of varied content and design, which meant there were subtle differences in the definition of cannabis use between studies. The inconsistent nature by which cannabis use is classified is a key limitation of many of the studies and may have had an effect on our results. For example, some studies referred to lifetime use as any previous use; therefore, this may have included people with previous heavy cannabis use as well as people who have tried it just once.

The majority of studies were rated as medium quality, with only one high‐quality study included in the analyses. The major source of bias related to exposure measurement as many studies did not use an objective method to classify cannabis use (such as blood/urine testing). Due to the classification of cannabis as an illicit substance in many countries, individuals may have been reluctant to admit use, leading to underreporting among both the UHR and control comparator groups. Another source of bias was that many studies also failed to control for confounding variables, such as age, gender, use of other substances and frequency of cannabis use. As mentioned previously, we were therefore, unable to control for other substances in our meta‐analysis. Cannabis users are more likely to use other substances; therefore, comorbid substance use may have accounted for higher rates of UTC/suspiciousness. We also could not control for the strength or frequency of cannabis use, and the last time a person used cannabis.

## Concluding remarks

Ultra high risk individuals have high rates of cannabis use and abuse which are significantly higher than HCs. Among UHR individuals, cannabis users have more severe unusual thought content and suspiciousness compared to non‐cannabis users. The UHR phase represents an important opportunity to intervene, and targeting substance use at this stage may have significant benefits to an individual's long‐term outcome. Clinicians should be aware of comorbid substance use disorders in young people at UHR for psychosis, and reduction in substance use should be a priority in youth mental health services.

## Supporting information


**Appendix S1.** Quality assessment and meta‐analyses outputs.
