# Supplementary material for: Cannabis use and symptom severity in individuals at ultra high risk for psychosis: a meta‐analysis
Source: Acta Psychiatr Scand. 2017 Feb 7;136(1):5–15. doi: 10.1111/acps.12699 (PMC5484316; doi:10.1111/acps.12699)

**Newcastle Ottawa Quality Assessment**

| **Study Name** | **UHR v HC** | |
| --- | --- | --- |
|  | **NOS score** | **Quality** |
| Addington et al. 2012 | 8 | High |
| Auther et al. 2012 | 6 | Medium |
| Bloemen et al. 2010 | 6 | Medium |
| Buchy et al. 2015 | 6 | Medium |
| Hagenmuller et al. 2016 | 5 | Medium |
| Pruessner et al. 2011 | 6 | Medium |
| Russo et al. 2014 | 6 | Medium |
| Stojanovic et al. 2014 | 6 | Medium |
| **Study Name** | **UHR Cannabis v non-cannabis users** | |
| Auther et al. 2012 | 4 | Medium |
| Auther et al. 2015 | 7 | Medium |
| Bugra et al. 2013 | 7 | Medium |
| Corcoran et al. 2008 | 5 | Medium |
| Dragt et al. 2012 | 4 | Medium |
| Gill et al. 2015 | 4 | Medium |
| Mizrahi et al. 2014 | 7 | Medium |
| Van Tricht et al. 2013 | 5 | Medium |

**Meta-analysis outputs**

Lifetime cannabis use (UHR individuals)


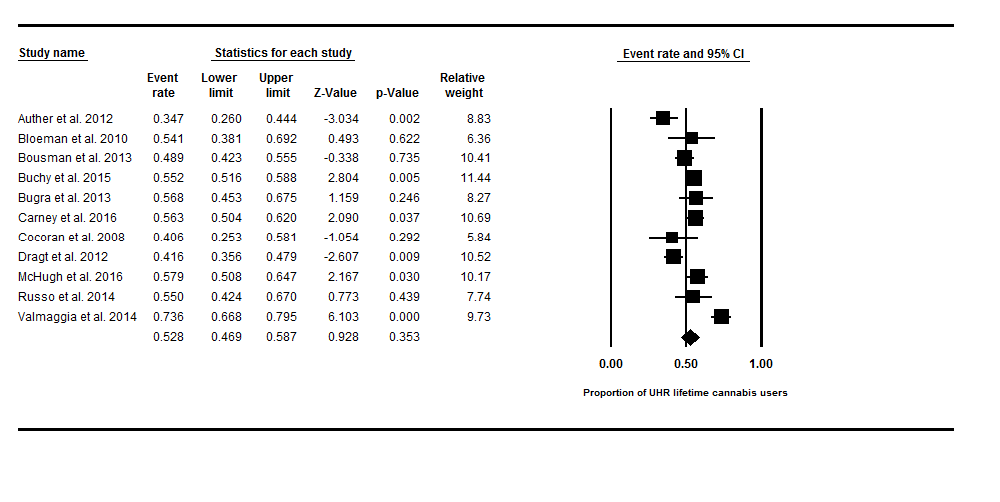


Current cannabis use disorders (UHR individuals)


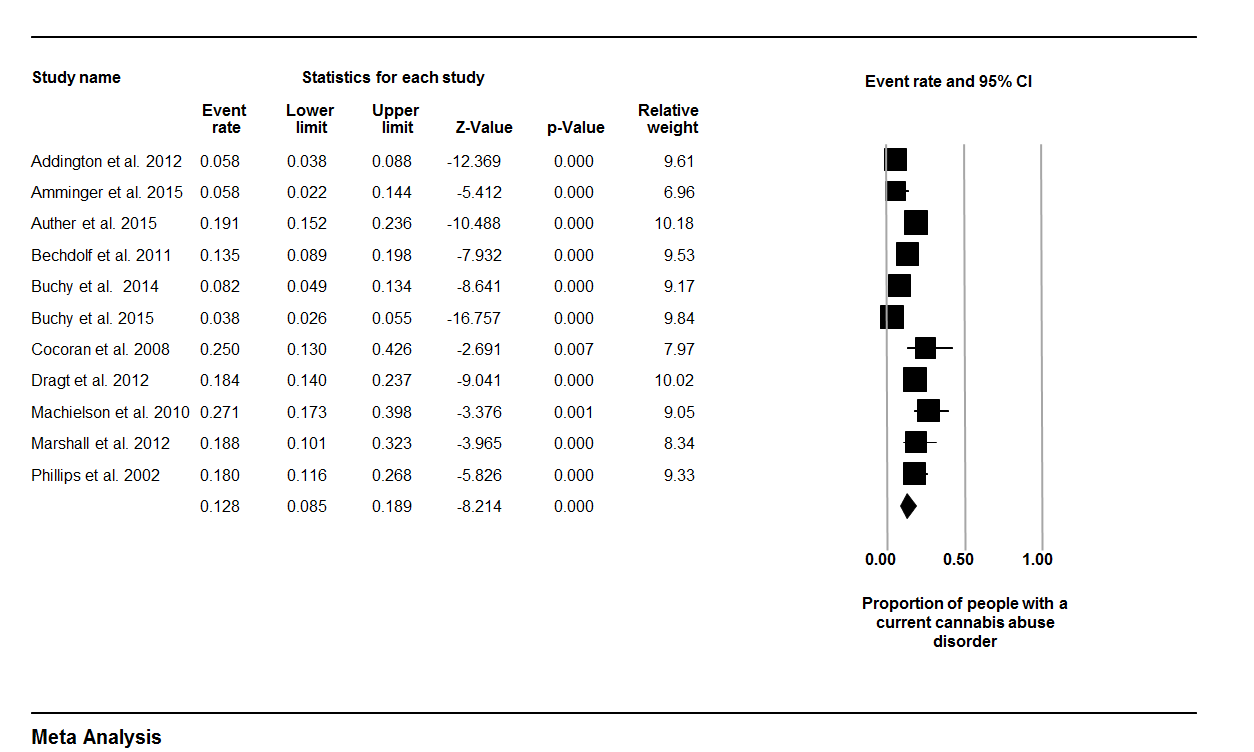


**UHR v Healthy Controls**


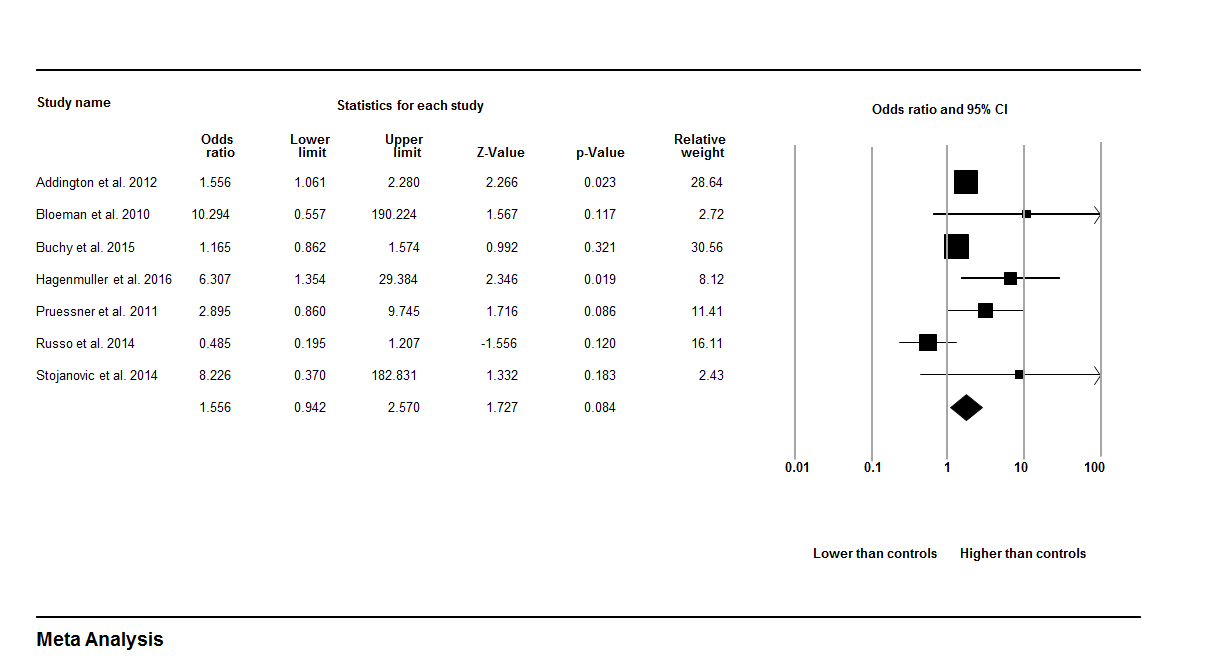
Current cannabis use

Lifetime cannabis use


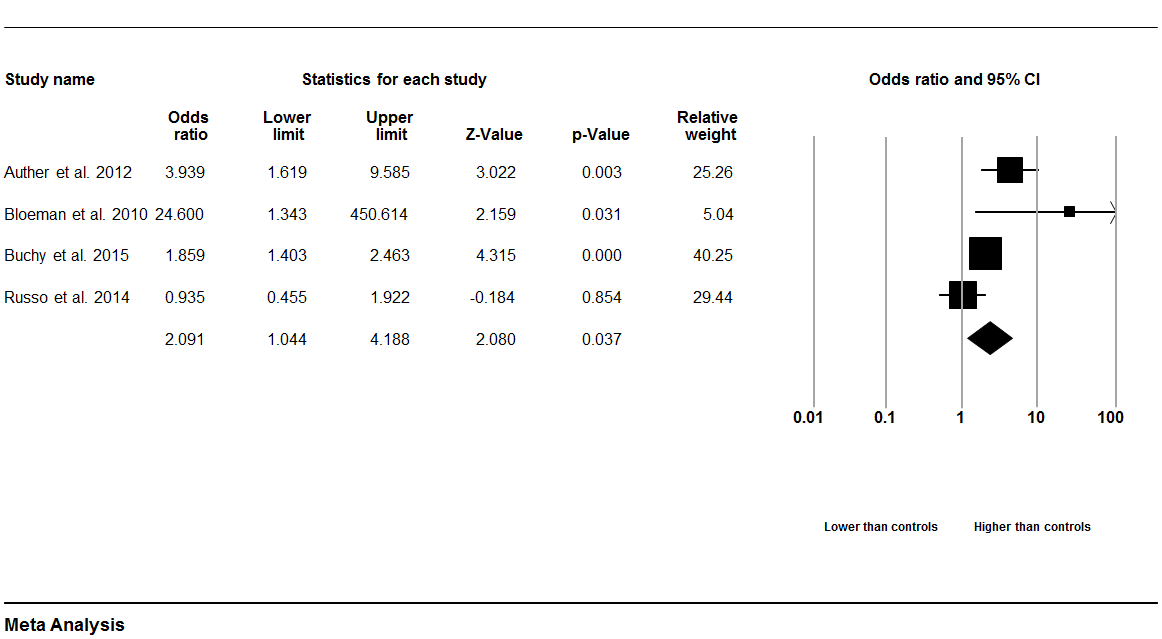


CUD


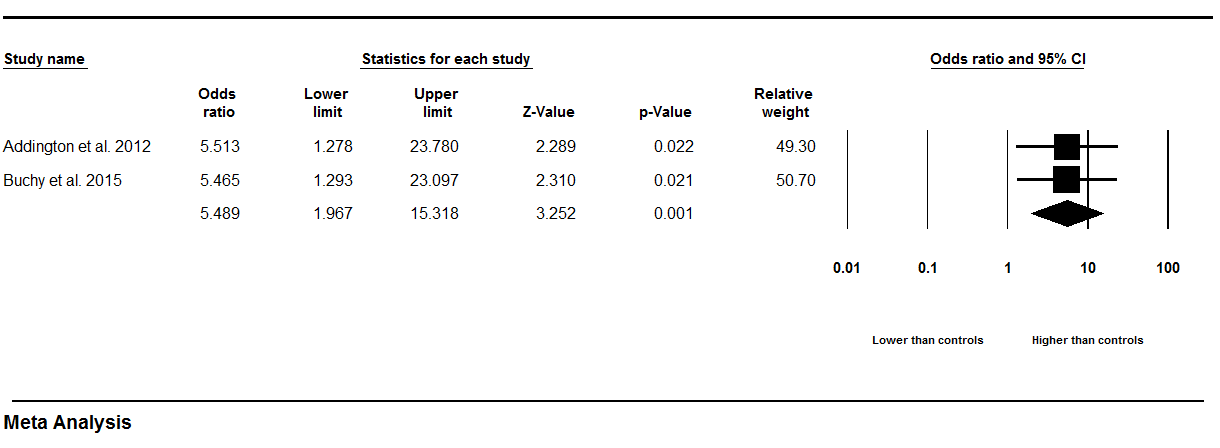


**UHR cannabis users compared with UHR non-cannabis users**

Total Positive Symptoms


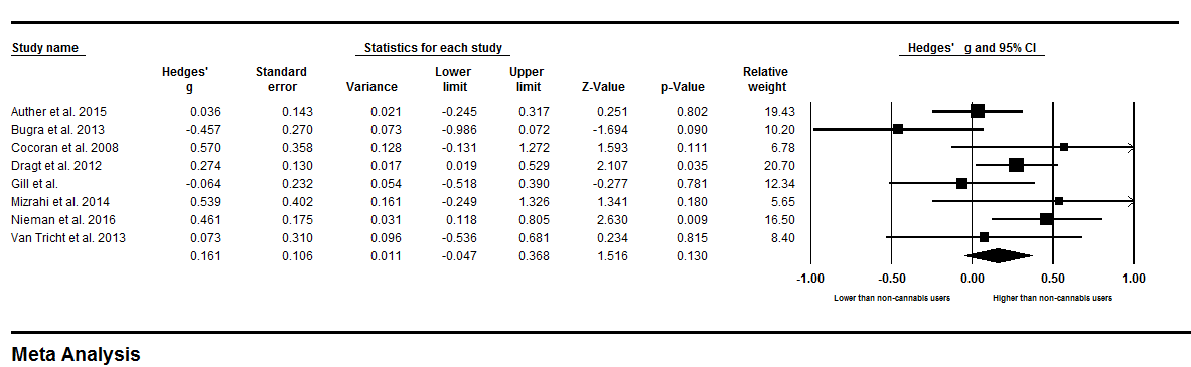


Disorganised speech
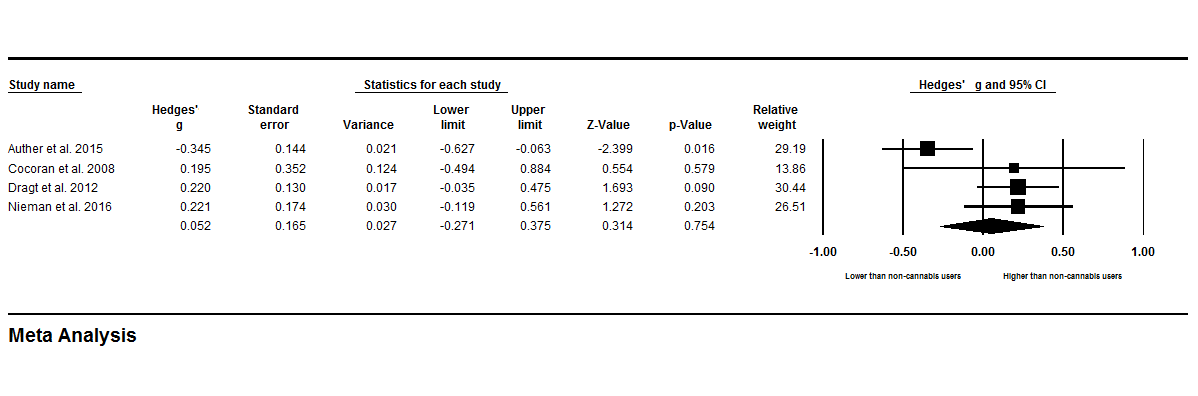


Perceptual abnormalities


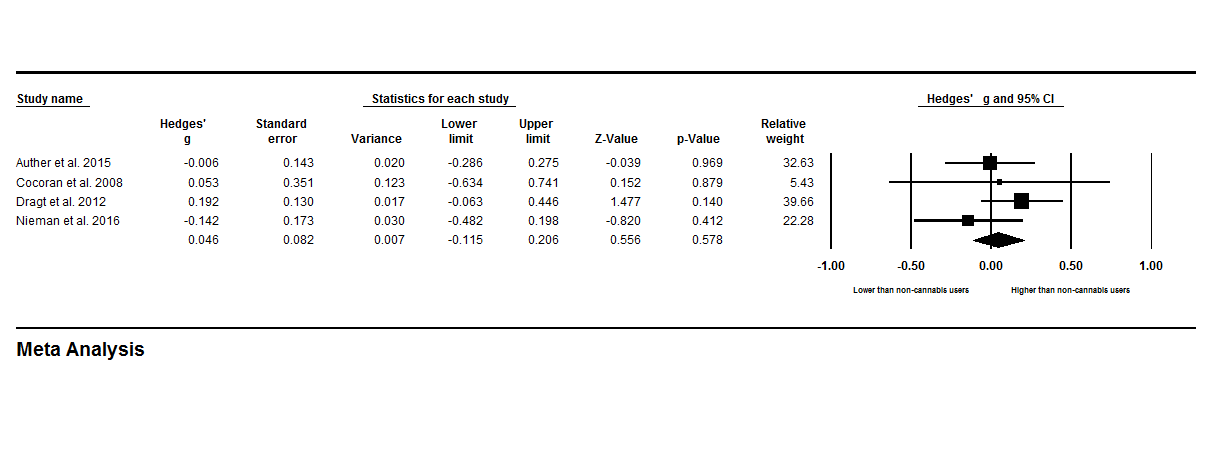


Unusual thought content


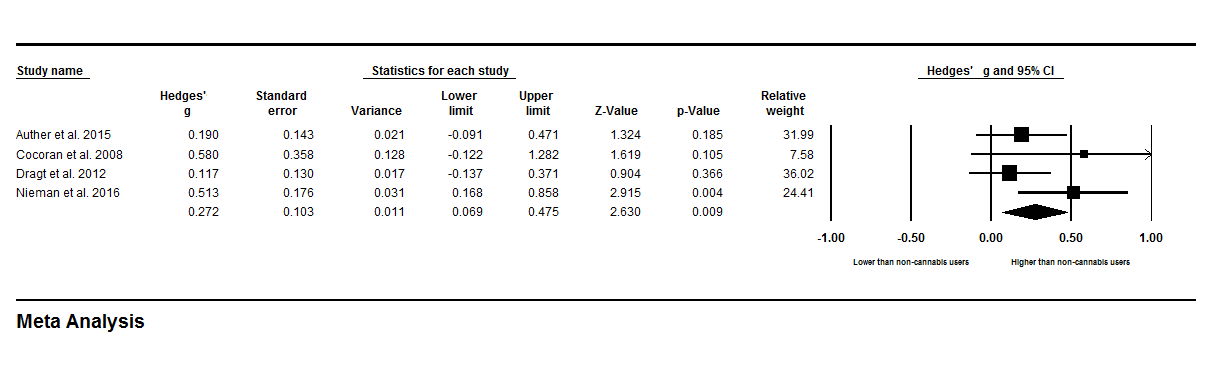


Suspiciousness


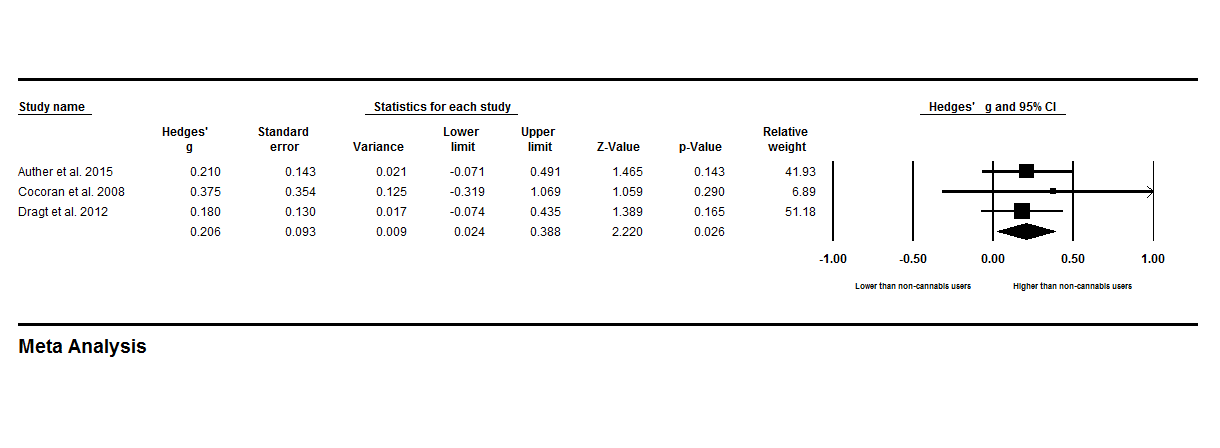


Grandiosity


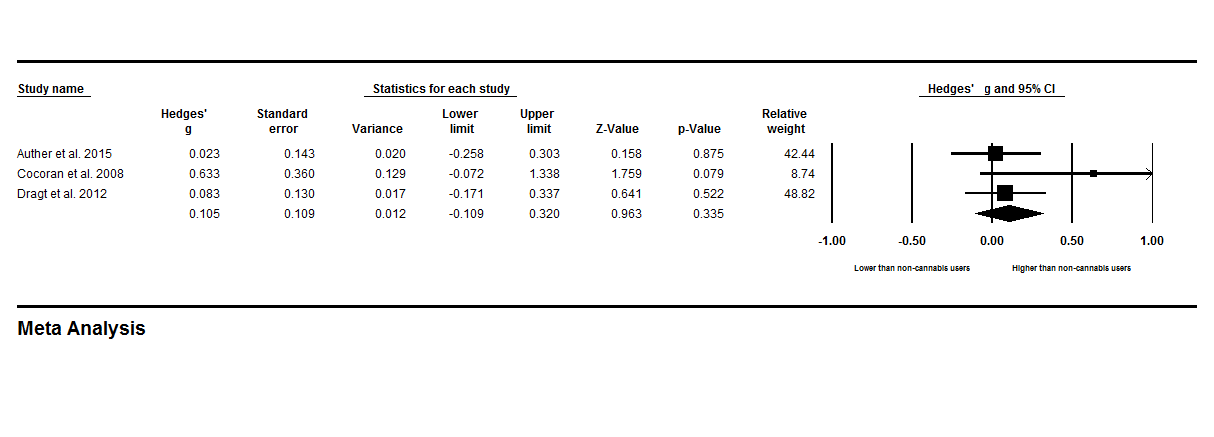


Total Negative Symptoms


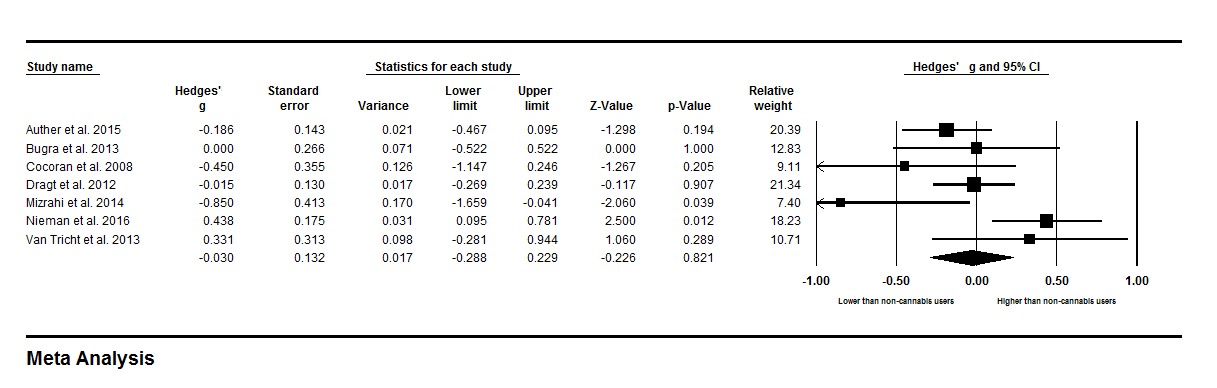

Supplement: Supplementary file 1 — Appendix S1. Quality assessment and meta‐analyses outputs. [file ACPS-136-5-s001.docx]
